# Bronchiolitis obliterans organizing pneumonia: Pathogenesis, clinical features, imaging and therapy review

**DOI:** 10.4103/1817-1737.39641

**Published:** 2008

**Authors:** Al-Ghanem Sara, Al-Jahdali Hamdan, Bamefleh Hanaa, Khan Ali Nawaz

**Affiliations:** *Department of Radiology, King Fahad National Guard Hospital, King Abdulaziz Medical City, Riyadh, Saudi Arabia*

**Keywords:** Bronchiolitis, cryptogenic organizing pneumonia, organizing pneumonia

## Abstract

Bronchiolitis obliterans organizing pneumonia (BOOP) was first described in the early 1980s as a clinicopathologic syndrome characterized symptomatically by subacute or chronic respiratory illness and histopathologically by the presence of granulation tissue in the bronchiolar lumen, alveolar ducts and some alveoli, associated with a variable degree of interstitial and airspace infiltration by mononuclear cells and foamy macrophages. Persons of all ages can be affected. Dry cough and shortness of breath of 2 weeks to 2 months in duration usually characterizes BOOP. Symptoms persist despite antibiotic therapy. On imaging, air space consolidation can be indistinguishable from chronic eosinophilic pneumonia (CEP), interstitial pneumonitis (acute, nonspecific and usual interstitial pneumonitis, neoplasm, inflammation and infection). The definitive diagnosis is achieved by tissue biopsy. Patients with BOOP respond favorably to treatment with steroids.

BOOP is a distinct clinicopathological entity with clinical, imaging and prognostic features different from those of obliterative bronchiolitis and usual interstitial pneumonia/idiopathic pulmonary fibrosis (UIP/IPF).[1–6] BOOP is characterized by the presence of granulation tissue in the bronchiolar lumen, alveolar ducts and some alveoli, associated with a variable degree of interstitial and airspace infiltration by mononuclear cells and foamy macrophages. BOOP is differentiated from organizing pneumonia, which is defined by the presence of granulation tissue in the distal air spaces; but when associated with granulation tissue in the bronchiolar lumen, organizing pneumonia is qualified by the term bronchiolitis obliterans (BO). Hence the term ‘bronchiolitis obliterans organizing pneumonia’ is used.[2,5–6]

## Pathogenesis of BOOP

**Figure 1:**
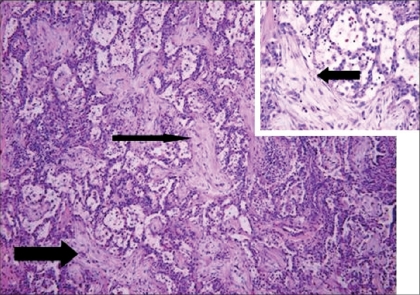
Case of idiopathic BOOP, shown on low power [magnification × 10] - pale staining areas of elongated branching fibrosis, involving bronchiolar lumen and peribronchial airspaces [solid arrow]. The alveolar septae [inset] shows mild chronic inflammation

**Figure 2:**
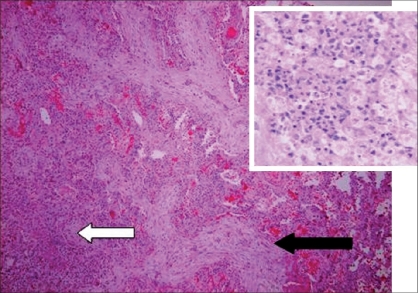
Case of BOOP with associated abscess. The pale elongated, serpiginous branching fibrous plugs in the alveolar spaces are demonstrated by the solid arrow. The abscess area is demonstrated by transparent arrow magnified × 40 in the inset. HandE stain, magnification × 10

Cytological profile of bronchoalveolar lavage in BOOP reveals a mixed cell pattern with an increase in lymphocytes (20-40%), and lymphocytes in patients with BOOP help differentiate the condition from parenchymal pulmonary disease. Neutrophils (10%), eosinophils (5%), mast cells, foamy macrophages and occasional plasma cells increase in patients with IPF. The number of eosinophils is increased significantly in patients with CEP, with a small overlap with BOOP. The CD4/CD8 ratio is decreased, but the percentage of CD57+ cells is within the reference range.[7] Activation of T cells is increased in terms of human leukocyte antigen-DR expression; and occasionally, interleukin-2 receptor (CD25) is also expressed. These findings are similar to those in extrinsic allergic alveolitis except that CD25 expression is always within the reference range in patients with BOOP and levels of CD57+ cells are always increased in extrinsic allergic alveolitis.[7] Other features include central clusters of mononuclear inflammatory cells, possibly found in the intraluminal polyps; chronic inflammation in the walls of the surrounding alveoli with reactive type II cells; increased foamy macrophages in the alveoli; and preserved lung architecture.[5] Several causes of BOOP have been recorded but most cases are idiopathic. BOOP has also been reported as a secondary phenomenon in several other clinical settings. Many lung pathologies are associated with BOOP, the BOOP process is histological and clinical course is that of the underlying primary pathology [Figures 1 and 2].

The pathogenesis of BOOP is that of an inflammatory lung disease rather than a fibrosing process such as in UIP/IPF. However, the inflammatory response in BOOP appears to be different from that in other pulmonary inflammatory disorders such as COPD, asthma and granulomatous lung disease. These differences are important because treatment directed towards one type of inflammatory response might not be effective against another type.[8] BOOP responds well to corticosteroid therapy, while UIP/IPF usually does not. There is a new fibromyxoid connective formation in BOOP, as well as in UIP/IPF; but this process can be fully reversed with corticosteroid therapy in BOOP but not in UIP/IPF [Figures 1 and 2]. This phenomenon is not completely understood. However, it is known that the newly formed fibromyxoid connective tissue causes destruction of the lung interstitium in UIP/IPF. There is extensive capillarization of the airway fibromyxoid tissue in BOOP but not in UIP/IPF. It is therefore postulated that vascular growth factors may cause apoptosis in BOOP but not in UIP/IPF, which may be responsible for the resolution of fibromyxoid connective tissue in BOOP.[9–11]

## Clinical Findings

Persons of all ages can be affected. Approximately 50% of patients present with influenza-like illness followed by a short illness of approximately few months′ duration characterized by a persistent nonproductive cough, effort dyspnea, low-grade pyrexia, malaise and weight loss. In one series 94% had symptoms of cough, fever or dyspnea. Less common symptoms include pleuritic chest pain and hemoptysis. Associated collagen vascular disorder is found in 16% and inhalation exposure to toxins in 17%.[12,13] Clinical examination of the thorax reveals fine, dry lung crepitations in the majority of patients. Clubbing is unusual. The ESR is usually elevated, and pulmonary function tests show a restrictive pattern. The diffusing capacity is reduced, the resting alveolar arterial oxygen gradient is widened and exercise-related hypoxemia is present. Conventional chest radiographs revealed that bilateral patchy infiltrates were seen most frequently in 68%, followed by small linear opacities in 15%, both patchy infiltrates and reticulonodular opacities in 12% and reticulonodular opacities in 6%.[14]

A third of patients with BOOP treated for less than a year may have recurrence of the disease. Nevertheless, BOOP can be effectively treated a second and third time with the previously effective dosage level of prednisone.[1] Nonresponsive BOOP may be secondary or may be associated with other pathology; or may represent a combination of BOOP and UIP/IPF, where the associated fibrotic process does not respond to corticosteroids.

In idiopathic BOOP, cough and dyspnea are usually mild; rare presentation with hemoptysis and respiratory distress has been reported.[15–16] Radiographic features with nodules, solitary nodule, pneumothorax and pleural effusion have been described.[13,17–19]

The rare rapidly progressive form of BOOP has an extremely poor prognosis.[20–21] This form can occur in a previously healthy individual or can be associated with other systemic illness. The course of the disease can be galloping, with 1 to 3 days of symptoms and acute respiratory failure.[22] In fatal cases, an underlying fibrotic process has been implicated. Most fatal cases have a secondary form of BOOP. The rapidly progressive primary form of BOOP seems to have a better prognosis.[20–21] A rapidly progressive BOOP can be indistinguishable from acute interstitial pneumonitis (AIP) on clinical grounds.[23–25] Urgent tissue sampling may lead to urgent introduction of corticosteroids for primary BOOP, which might improve survival.[21]

Focal lung nodules in the idiopathic form of BOOP are clinically important as they may mimic lung cancer.[16,18,26–29] Some focal nodular lesions may progress to the typical bilateral process of idiopathic BOOP but most do not. Surgical resection may result in a cure. Multiple nodular lesions can also occur, and most regress spontaneously or following treatment with corticosteroids.[28–29] Thus BOOP should be considered when multiple large nodular lesions have chest CT findings of air bronchogram, irregular margins, broad pleural tags, parenchymal bands or subpleural lines. In patients with multiple BOOP nodules, the mode of presentation is pleuritic type chest pain in approximately 50% patients. The nodular type of BOOP may be related to reports of spontaneous resolution of lung metastases.[30] It has been suggested that reports of spontaneous regression of lung metastases have decreased in recent years with increasing reliance on tissue diagnosis and with confirmation of the fact that the nodular lesions indeed represent nodular BOOP.

BOOP can follow all types of pneumonias when symptoms and radiographic changes persist despite an initial improvement. Thus the pneumonic process organizes into BOOP. This distinction is important to make as these patients invariably respond well to corticosteroids.[11,13,28,30–39]

A variety of drugs have been associated with BOOP.[11,13,37–43] Cases of phenytoin-associated BOOP and carbamazepine-induced lupus erythematosus and associated BOOP responded rapidly to corticosteroids.[38–39] A case of ticlopidine-associated BOOP resolved following the withdrawal of the offending agent.[42]

BOOP has been reported in association with all connective tissue disorders, which respond well to corticosteroids unlike lung fibrotic changes that may occur in connective tissue. [43–54] However, a case of corticosteroid-resistant BOOP in association with dermatomyositis has been reported that improved when cyclophosphamide was added to the patient's regimen.[47]

BOOP has been reported in association with organ transplantation, especially with bone marrow transplant.[52–55] BOOP may follow lung transplantation, occurring at 1 to 10 months following surgery, and usually accompanies acute rejection.[53–55] BOOP lesions respond well to treatment if the underlying acute rejection is successfully treated. CMV pneumonia-associated BOOP can occur with lung transplantation, which usually responds well to corticosteroids.[55] BOOP associated with renal transplantation has been described in only one patient, which responded quickly with an increase in dose of corticosteroids.[12]

BOOP associated with radiotherapy usually occurs in patients receiving radiotherapy for breast cancer. The radiographic findings are those of peripheral patchy or alveolar infiltrates, often outside the radiation field, responding rapidly to corticosteroids.[53,56–61].

Many industrial toxins and environmental pollutants have been associated with BOOP. [59–63]

BOOP is associated with a variety of unrelated miscellaneous conditions [Table 1], including: essential mixed cryoglobulinemia, myelodysplastic syndrome, interstitial cystitis, chronic thyroiditis, sarcoidosis, alcoholic cirrhosis and, in England, seasonal syndrome with cholestasis. There has been one report of BOOP during menstrual and pregnancy-related inflammatory bowel disease. The BOOP lesion might be associated with lymphoma/leukemia and other neoplastic processes. BOOP has also been reported in primary biliary cirrhosis and after coronary artery bypass graft surgery, Evans syndrome and chronic sinusitis, lung cancer, lung atelectasis, asthma, cystic fibrosis, secondary amyloidosis, Sweet syndrome, idiopathic thrombocytopenic purpura and Fabry disease. A case report of ITP-associated BOOP spontaneously resolved after suppression of cyclosporine and positive serology for Epstein-Barr virus.[13,62–103]

**Table 1:** Causes of bronchiolitis obliterans with organizing pneumonia

Idiopathic bronchiolitis obliterans with organizing pneumonia
Post-infection
Bacterial infection:
*Mycoplasma pneumoniae*
*Staphylococcus aureus*
*Streptococcus pneumoniae*
*Chlamydia pneumoniae*
*Pseudomonas aeruginosa*
*Legionella pneumophila*
*Nocardia asteroides*
*Coxiella burnetii*
*Serratia marcescens*
Viral infection
Herpes virus
Influenza virus,
Parainfluenza virus
Human immunodeficiency virus
Drugs:
Antiobiotics;
Sulfasalzine,
Cephalosporin
Sulfamethoxypyridazine
Amphotericin
Acebutolol
Sotalol
Amiodarone
Bleomycin
Busulphan
Methotrexate
Carbamazepine
Cocaine
Gold salts
Interferon alpha
Phenytoin
Tacrolimus
Ticlopidine
Vinabarbital-aprobarbital
Connective tissue/immunologic disease
lupus erythematosis
rheumatoid arthritis
sjogren syndrome
polymyositis/dermatomycitis
Behcet disease
Polymylagia rheumatica
Ankylosing spondolitis
Sweet syndrome
Essential mixed cryoglobulinemia
Common variable immunodefincey syndrome
Organ transplantation:
Lung, renal, bone marrow transplant
Radiotherapy
Environmental
Textile printing dye
House fire
Miscellaneous:
Inflammatory bowel disease
Cancer (solid and hematological)
Myelodysplastic syndrome

## The Middle Eastern Experience

To compare clinico-radiological features and outcomes in Saudi Arabia with international experience in patients with BOOP, a retrospective study was carried out in three large hospitals in Riyadh. The clinical profile in 20 Saudi patients was similar to that described in the world literature. The outcomes were favorable except in patients with underlying systemic disease.[104]

## Imaging Findings

Plain radiograph findings include the following:[7,26,32,105–108]

- Bilateral or unilateral patchy alveolar airspace consolidation is revealed, often subpleural and peribronchial in location and mainly in the lower zones.
- Generally, the infiltrates gradually enlarge from their original size or new infiltrates appear.
- Consolidation is often nonsegmental and varies from 2 to 6 cm in diameter.
- Cavitary BOOP that mimics tuberculosis and cavitating opacity after lung transplantation has been reported.
- An air bronchogram may be present.
- Nodules 3-5 mm in diameter are seen in approximately one half of patients; nodules may be migratory.
- Basal linear opacities are linked to a poorer prognosis.
- Unilateral focal or lobar consolidation occurs in 5-31% of patients.
- Miliary pattern is rare.
- Pleural thickening occurs but pleural effusions are rare.

## HRCT

**Figure 3:**
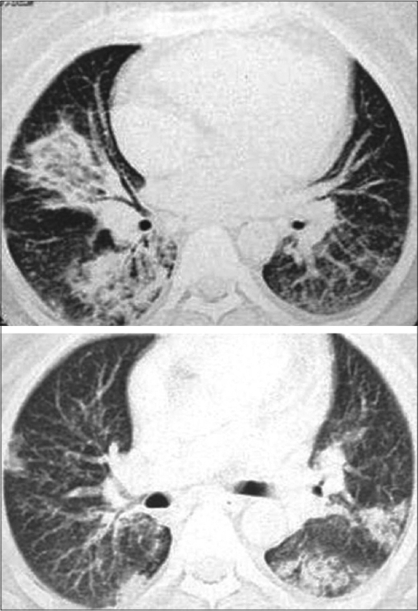
BOOP presenting as airspace and nodular opacities (L). Typical picture of BOOP with peripheral bilateral airspace opacities, predominantly at the bases (R).

**Figure 4:**
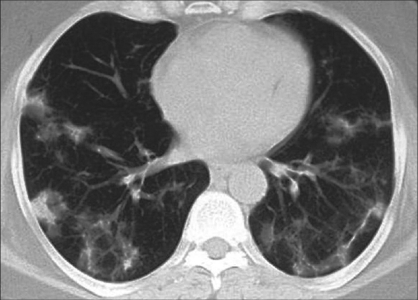
Multiple bilateral airspace and interstitial patchy opacities

**Figure 5:**
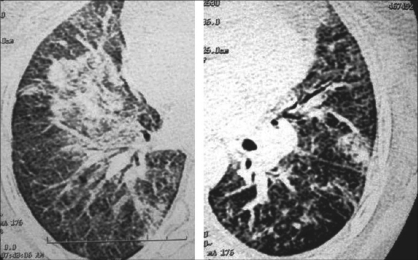
Left: endobronchial and acinar filling with tree-in-bud appearance with mild interstitial thickening. Right: Interstitial thickening and airspace opacities as a presentation of BOOP

**Figure 6:**
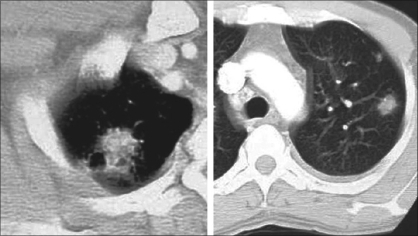
BOOP presenting as a nodule with partial spiculation (R) and peripheral nodules (L)

HRCT findings have been well described in BOOP[4,26–28,68,72,108–127]:

- Neither conventional radiographs nor CT findings are specific to BOOP and are seen in a variety of lung-infective, inflammatory and neoplastic processes. However, CT is more sensitive than chest radiography in the assessment of disease pattern and distribution of disease. CT is superior in determining the biopsy site; therefore, HRCT is usually performed prior to lung biopsy.
- Early on, clinical and chest radiographic findings of various types of interstitial pneumonitis and BOOP may be indistinguishable; however, HRCT findings of AIP and BOOP may be different. Traction bronchiectasis, interlobular septal thickening and intralobular septal thickening are significantly more prevalent in patients with UIP and nip to a less extent than in patients with BOOP, whereas parenchymal nodules and peripheral distribution are more prevalent in BOOP. Areas with ground-glass attenuation, airspace consolidation and architectural distortion are common in both interstitial pneumonitis (IP) and BOOP.
- Patchy ground-glass opacities in a subpleural and/or peribronchovascular distribution (80%).
- Bilateral basal airspace consolidation (71%) [Figures 3 and 4].
- Bronchial wall thickening and cylindrical bronchial dilatation in areas of air bronchogram (71%) [Figure 3].
- Centrilobular nodules 3-5 mm in diameter in approximately one half of patients [Figure 3].
- Mild mediastinal lymphadenopathy (27%).
- Small nodular opacities measuring 1-10 mm in diameter, typically ill defined (50%).
- Cavitating lung mass (rare).
- Pleural effusions occur in approximately one-third of patients.
- Less frequent imaging findings include single or multiple focal lesions, parenchymal consolidations with peribronchovascular distribution [Figure 6]. The ‘atoll sign’ seen as a central area of ground-glass-like density and peripheral area of consolidation, poorly defined micronodular lesions, linear and band-like opacities, subpleural opacities that can have disposition parallel or perpendicular in relation to the pleura, perilobular pattern characterized by thickening of the interlobular septa associated with reticular pattern and progressive fibrotic pattern characterized by irregular thickening of the interlobular septa with associated ground-glass-like appearance and consolidations [Figure 5].

## Other imaging findings

An early report of the value of gadolinium-enhanced MRI in the evaluation of disease activity in BOOP showed promising results. In this report, 14 out of 17 patients with active disease showed enhancing lesions on T1-weighted breath-hold gadolinium-enhanced MRI. The white lung sign is an uncommon finding in pulmonary consolidations evaluated with heavily T2-weighted sequences. However, the sign is usually negative in patients with BOOP, but it was found to be positive in 5 of 5 patients with bronchoalveolar carcinoma in one study. Thus, MRI has a potential role in the differential diagnosis of BOOP.[113–114] At present, MRI has no diagnostic role in BOOP, but it may have a role in the follow-up imaging in patients with BOOP to assess the treatment response or disease activity. Ultrasonography is useful in the detection and characterization of pleural effusion and in the guidance of pleural interventions. A recent study has shown that patients with an enhanced 18F- FDG accumulation reflect the degree of disease activity in BOOP.[128]

## Diagnosing BOOP

Lung biopsy continues to be the preferred method for establishing the diagnosis. The multitude of interstitial lung diseases pose a diagnostic dilemma; however, the process may be made simpler by reviewing the patient's history, through specific serologic tests, by bronchoalveolar lavage, transbronchial biopsy, biopsy of extrathoracic tissues or open-lung biopsy. Conventional radiography and HRCT serve as a guide for further investigation and the site of lung biopsy. The lack of honeycombing or an irregular reticular pattern in BOOP may help to differentiate BOOP from other interstitial lung diseases. Lobar consolidation may be mistaken for lung malignancy, while identical peripheral airspace consolidation can be seen in CEP and BOOP. Whereas CEP has a predominant upper-lobe involvement, the consolidation in BOOP involves the lower zones to a greater degree; though some patients have pathologic features of both CEP and BOOP. Most patients with BOOP require open-lung biopsy for diagnosis. However, some evidence suggests that combining the cytologic bronchoalveolar lavage and histologic transbronchial lung biopsy data obtained during a fiberoptic procedure appears to be an effective method for initially investigating BOOP that presents with patchy radiographic shadows. Percutaneous lung biopsy has been used in a few patients; but on the whole, it appears to be inadequate. The video-assisted thoracoscopic procedure has become the established technique. In a study in which 12 of 49 patients underwent video-assisted thoracoscopic procedure for interstitial lung disease, the mean length of the operation was 45 min, the chest tube was inserted for 1.3 days, there were no deaths, there were no re-explorations and none were converted to an open thoracotomy.[129–130]

## Differential Diagnosis

Mimics of BOOP include collagen vascular disease, lung metastases [Figure 6], infective pneumonias, Wegener granulomatosis, eosinophilic pneumonia, primary bronchogenic neoplasm and tuberculosis. In one report, two patients with subacute symptoms and signs compatible with pulmonary tuberculosis had cavitary infiltrates in the right upper lobe, as demonstrated on chest radiographs. Histologic analysis of specimens from both patients yielded typical histologic features of BOOP.[108]

Four children following chemotherapy for malignant disease developed pulmonary infiltrates, and solitary nodules on imaging led to open-lung biopsy. Histologic diagnosis was that of BOOP.[98]

## IP vs. BOOP

The early clinical and radiographic findings of IP are often similar to those of BOOP. Differentiation is important as IP carries a poor prognosis. Analysis of certain HRCT findings has shown that traction bronchiectasis, interlobular septal thickening and intralobular reticular proliferation are more prevalent in UIP than in BOOP. Lung parenchymal nodules and peripheral distribution are more prevalent in BOOP than in IP. Areas with ground-glass attenuation, airspace consolidation and architectural distortion were common in both IP and BOOP. Thus, when differentiating BOOP from IP, special consideration should be given to the aforementioned radiographic features.[131–132]

To assess the role of chest radiography in the differential diagnosis of BOOP and UIP, Muller NL *et al.* chest radiography, clinical information and pulmonary function data were reviewed, without knowledge of the pathologic diagnosis. The clinical symptoms of BOOP were similar to those of UIP, although the duration of symptoms was longer in UIP and the prevalence of systemic symptoms was higher in BOOP. The physical findings were similar except that finger clubbing was more common in patients with UIP than in those with BOOP. No significant difference in lung volumes, flows or diffusing capacity was recorded. The chest radiograph showed distinguishing features between UIP and BOOP in the majority of patients. The most characteristic radiologic finding in BOOP was the presence of patchy areas of airspace consolidation.[132]

## Treatment of BOOP

BOOP may resolve spontaneously; however, corticosteroids are the current standard treatment. The majority of patients with BOOP recover with treatment, symptoms resolving within days or weeks. Similarly the radiographic findings show improvement in 50-86% of patients; however, in a minority of patients, the disease may persist. Approximately 30% of the patients experience relapse upon withdrawal of treatment. Patients with asymptomatic mass lesions or nonprogressive disease can be observed and treated at a later time if needed. There is no consensus regarding the optimal doses of prednisone and optimal treatment duration. The dosage is generally 0.75 mg/kg/day for 1 to 3 months, then 0.50 mg/kg mg/day for 3 months, then 10 to 20 mg/day or every other day for a total of 1 year. Every-other-day scheduling can be successfully used for this disorder. A shorter 6-month course may be sufficient in certain situations. However, this duration can extend up to 12 months or even longer due to relapses. A total and permanent recovery is seen in most patients, but it is also dependent on the cause or associated systemic disorders. Anecdotally, erythromycin, inhaled triamcinolone, azathioprine, cyclosporin and cyclophosphamide have been used to treat BOOP.[19–21]

## Conclusion

BOOP is an important treatable inflammatory lung disease. Idiopathic BOOP has become an important differential of focal lung nodular lesions. Postpneumonia BOOP remains a treatable process. BOOP occurs in virtually all of the connective tissue disorders and generally responds to corticosteroids. In the setting of malignancy, it is vital to differentiate lung metastases from BOOP. Lung biopsy continues to be the preferred method for establishing a diagnosis.
